# Microtubule-Stabilizer Epothilone B Delays Anesthetic-Induced Unconsciousness in Rats

**DOI:** 10.1523/ENEURO.0291-24.2024

**Published:** 2024-08-27

**Authors:** Sana Khan, Yixiang Huang, Derin Timuçin, Shantelle Bailey, Sophia Lee, Jessica Lopes, Emeline Gaunce, Jasmine Mosberger, Michelle Zhan, Bothina Abdelrahman, Xiran Zeng, Michael C. Wiest

**Affiliations:** Neuroscience Department, Wellesley College, Wellesley, Massachusetts 01760

**Keywords:** consciousness, microtubules, neoadjuvant, orchestrated objective reduction, postoperative cognitive dysfunction, volatile anesthetics

## Abstract

Volatile anesthetics are currently believed to cause unconsciousness by acting on one or more molecular targets including neural ion channels, receptors, mitochondria, synaptic proteins, and cytoskeletal proteins. Anesthetic gases including isoflurane bind to cytoskeletal microtubules (MTs) and dampen their quantum optical effects, potentially contributing to causing unconsciousness. This possibility is supported by the finding that taxane chemotherapy consisting of MT-stabilizing drugs reduces the effectiveness of anesthesia during surgery in human cancer patients. In order to experimentally assess the contribution of MTs as functionally relevant targets of volatile anesthetics, we measured latencies to loss of righting reflex (LORR) under 4% isoflurane in male rats injected subcutaneously with vehicle or 0.75 mg/kg of the brain-penetrant MT–stabilizing drug epothilone B (epoB). EpoB-treated rats took an average of 69 s longer to become unconscious as measured by latency to LORR. This was a statistically significant difference corresponding to a standardized mean difference (Cohen's *d*) of 1.9, indicating a “large” normalized effect size. The effect could not be accounted for by tolerance from repeated exposure to isoflurane. Our results suggest that binding of the anesthetic gas isoflurane to MTs causes unconsciousness and loss of purposeful behavior in rats (and presumably humans and other animals). This finding is predicted by models that posit consciousness as a property of a quantum physical state of neural MTs.

## Graphical abstract

**Figure:**
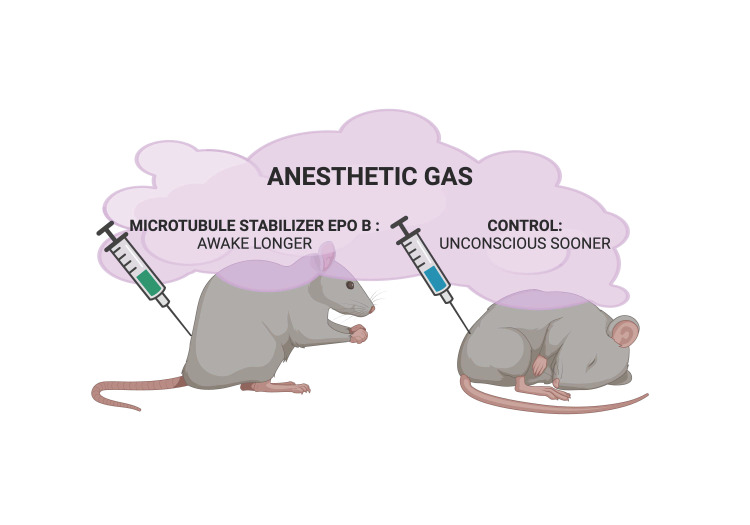
Visual Abstract

## Significance Statement

Our study establishes that action on intracellular microtubules (MTs) is the mechanism, or one of the mechanisms, by which the inhalational anesthetic gas isoflurane induces unconsciousness in rats. This finding has potential clinical implications for understanding how taxane chemotherapy interferes with anesthesia in humans and more broadly for avoiding anesthesia failures during surgery. Our results are also theoretically important because they provide support for MT-based theories of anesthetic action and consciousness.

## Introduction

Volatile anesthetics reversibly abolish consciousness or motility in animals, plants, and single-celled organisms (Kelz and Mashour, 2019; Yokawa et al., 2019). For humans, they are a medical miracle that we have been benefiting from for over 150 years, but the precise molecular mechanisms by which these molecules reversibly abolish consciousness remain elusive (Eger et al., 2008; Hemmings et al., 2019; Kelz and Mashour, 2019; Mashour, 2024). The functionally relevant molecular targets for causing unconsciousness are believed to be one or a combination of neural ion channels, receptors, mitochondria, synaptic proteins, and cytoskeletal proteins.

The Meyer–Overton correlation refers to the venerable finding that the anesthetic potency of chemically diverse anesthetic molecules is directly correlated with their solubility in lipids akin to olive oil (S. R. Hameroff, 2018; Kelz and Mashour, 2019). The possibility that general anesthesia might be explained by unitary action of all (or most) anesthetics on one target protein is supported by the Meyer–Overton correlation and the additivity of potencies of different anesthetics (Eger et al., 2008). Together these results suggest that anesthetics may act on a unitary site, via relatively nonspecific physical interactions (such as London/van der Waals forces between induced dipoles).

Cytoskeletal microtubules (MTs) have been considered as a candidate target of anesthetic action for over 50 years (Allison and Nunn, 1968; S. Hameroff, 1998). Other membrane receptor and ion channel proteins were ruled out as possible unitary targets by exhaustive studies culminating in Eger et al. (2008). However, MTs (composed of tubulin subunits) were not ruled out and remain a candidate for a unitary site of anesthetic action. MTs are the major components of the cytoskeleton in all cells, and they also play an essential role in cell reproduction—and aberrant cell reproduction in cancer—but in neurons, they have additional specialized roles in intracellular transport and neural plasticity (Kapitein and Hoogenraad, 2015). MTs have also been proposed to process information, encode memory, and mediate consciousness (S. R. Hameroff et al., 1982; S. Hameroff and Penrose, 1996; S. Hameroff, 2022). While classical models predict no direct role of MTs in neuronal membrane and synaptic signaling, Singh et al. (2021a) showed that MT activities do regulate axonal firing, for example, overriding membrane potentials. The orchestrated objective reduction (Orch OR) theory proposes that anesthesia directly blocks quantum effects in MTs necessary for consciousness (S. Hameroff and Penrose, 2014). Consistent with this hypothesis, volatile anesthetics do bind to cytoskeletal MTs (Pan et al., 2008) and dampen their quantum optical effects (Kalra et al., 2023), potentially contributing to causing unconsciousness.

This hypothesis is further supported by observations of resistance to anesthesia in human recipients of taxane chemotherapy. The taxanes are a class of MT-stabilizing drugs used to treat multiple forms of cancer (Varidaki et al., 2018). Recipients of taxane chemotherapy had a significantly elevated blood pressure response to incision during surgery for breast cancer and required significantly more opioid analgesic, as compared with patients who did not receive taxane chemotherapy (Linganna et al., 2015). This result suggests that binding to MTs contributes to inducing unconsciousness under volatile anesthetics, but on the other hand, there was no significant change in the heart rate or anesthetic delivery during surgery in the taxane group as compared with controls. Similarly, the variety of pathological profiles among patients, the variety of anesthetics and other drugs used, and the variety of taxane therapies used in that study make it difficult to draw firm or specific conclusions.

In order to quantitatively assess the functional relevance of MTs as one of the anesthetic targets contributing to loss of consciousness, we administered the MT-stabilizing drug epothilone B (epoB) to a group of rats and compared their times to fall unconscious under isoflurane anesthesia before and after epoB treatment.

## Materials and Methods

### Animals

All experimental procedures were performed in accordance with the Wellesley College animal care committee's regulations. A total of 12 male Long–Evans rats (Charles River Laboratories) were used in this study. Eight of these comprised our main experimental group for experiments with epoB. The other four animals were used for control experiments to estimate tolerance effects from repeated exposure to isoflurane in the absence of epoB treatment. At the start of the experiment, rats in the main experimental group (*N* = 8) were 2 months old and their weights ranged from 220 to 266 g. The rats from the additional “tolerance” group (*N* = 4) were 5 months old and had a weight range between 514 and 700 g at the start of their anesthesia sessions. Rats were housed in pairs in a dedicated animal care facility on a 12/12 h light/dark schedule (lights on at 6 A.M./off at 6 P.M.) with *ad libitum* access to food and water. Tails were marked with felt-tipped pens to identify individuals.

### Drug preparation and administration

Epothilones are a class of MT-stabilizing drugs used for cancer treatment. Epothilones are distinguished from older MT-stabilizing drugs by their better ability to penetrate the blood–brain barrier and affect the brain and spinal cord. This has led to investigations of their therapeutic effects in animal models of Alzheimer's disease and other tauopathies (Varidaki et al., 2018). Following previous work (Ruschel et al., 2015; Griffin et al., 2023), we prepared injections containing 0.75 mg/kg epoB or vehicle only. The vehicle was a 50/50% mixture of DMSO and saline. Injection volumes were <1 ml. (Griffin et al., 2023) compared with the effectiveness of intraperitoneal versus subcutaneous injections and found greater concentrations of the drug in the brain with subcutaneous injections. On this basis we administered epoB subcutaneously. Those authors also measured brain concentrations of epoB over time and found sustained levels of epoB in the brain through the 16 d period of their measurements. Note that known side effects of epoB include tiredness/weakness and fatigue (Sessa et al., 2007), but not hyperarousal, so epoB would not be expected to cause resistance to anesthesia through an off-target effect on arousal.

### Anesthesia sessions

Following standard practice in the field (Campagna et al., 2003; Icaza et al., 2009; McCarren et al., 2013), we used latency to loss of righting reflex (LORR) as a proxy for time to become unconscious. Rats were placed in an induction chamber and exposed to 4% isoflurane in oxygen (flow rate 1.5 L/min). The LORR latency was recorded when the rat was fully unconscious as assessed by turning it on its back. If it failed to place all four paws back on the ground within 30 s, the LORR latency was recorded. If it did right itself within 30 s, then LORR latency was recorded as the time when it ultimately did fail the LORR test. Subcutaneous injection of vehicle or epoB (as described below) for the main experimental group was performed after recording the LORR data. This means that the first day of epoB injection is the last day of the control (vehicle) condition, not the first day of the epoB condition. Our experimental design involved injections during every experimental session. To avoid stressing the rats before taking LORR measurements, which would likely introduce large uncontrolled variability into the data, we gave injections after isoflurane induction. This preserved the integrity of the LORR latency data but unfortunately did introduce large variability into the emergence (latency to regaining of righting response) measurements, which prevented our drawing any firm conclusions from those data.

### Blinding procedure and experimental schedule

To eliminate any possibility of experimenter bias, epoB and vehicle injections were prepared for each anesthesia session for each rat by two of us (Y.H. and M.C.W.) so that researchers taking the LORR latency measurements could not know whether the injections were epoB or vehicle. Moreover, they did not know when we would stop taking LORR latency measurements or whether we would provide additional doses of epoB after the first. All researchers were trained to use the identical procedure for determining LORR latency, and at least two researchers (both blind) were present for every experiment. Data were collected for up to 22 d after the first epoB injection. Six of eight rats were injected with epoB once, and all their other injections were vehicle (50/50% saline/DMSO). Two rats (I1 and I2) were given a second epoB injection on the 14th day after their first. These eight rats will be referred to as the “main experimental group.”

In a separate group of four rats from the eight taking part in the epoB experiments described above, we ran experiments to estimate the effect of repeated isoflurane anesthesia sessions on LORR latency. These four rats will be referred to as the “tolerance group.”

### Statistics

We used a within-subject experimental design to compare LORR latencies in the same rats before and after epoB treatment. To evaluate the difference between LORR latencies pre- and post-epoB injection, we first separately averaged LORR latencies from pre- and post-epoB sessions for each rat. A two-tailed permutation *t* test (*N* = 8 rats) was used to compare average LORR latencies in the two conditions. The results were considered to be significant if the *p* value was <0.05.

## Results

### Average LORR latency increased after epoB injection

In order to test whether isoflurane causes unconsciousness in part by binding to MTs, we compared latencies to LORR under 4% isoflurane in eight male Long–Evans rats before and after injection with 0.75 mg/kg epoB. Researchers taking the LORR latency measurements and administering injections were blind to the injection and drug condition of the animal.

**Figure 1.**
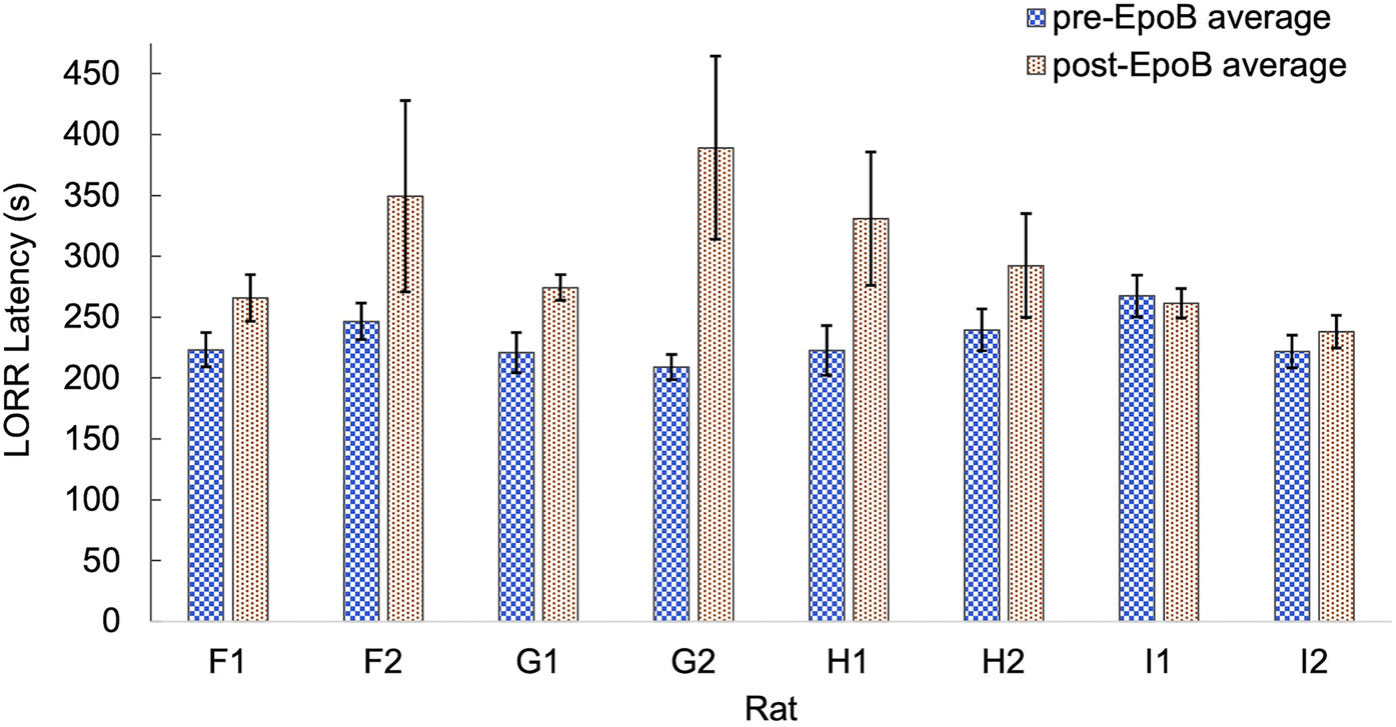
Average LORR latency for each rat in anesthesia sessions before and after epoB injection. Seven of eight rats show higher average LORR latency in the post-epoB injection condition. The numbers of sessions included in the pre- and post-epoB averages for each rat are shown in Table 1. Error bars represent standard errors computed using the variability across sessions. The same letter in two rats’ names indicates cage mates.

Figure 1 shows the average pre- and post-epoB LORR latencies (±SE) for each rat. Seven of the eight rats exhibited increased average LORR latencies in the epoB condition.

**Table 1.** Numbers of anesthesia sessions in our main data set in the pre-epoB and post-epoB conditions

Rat	Pre-epoB sessions	Post-epoB sessions
F1	4	10
F2	4	10
G1	6	8
G2	6	8
H1	5	9
H2	5	9
I1	3	11
I2	3	11

**Figure 2.**
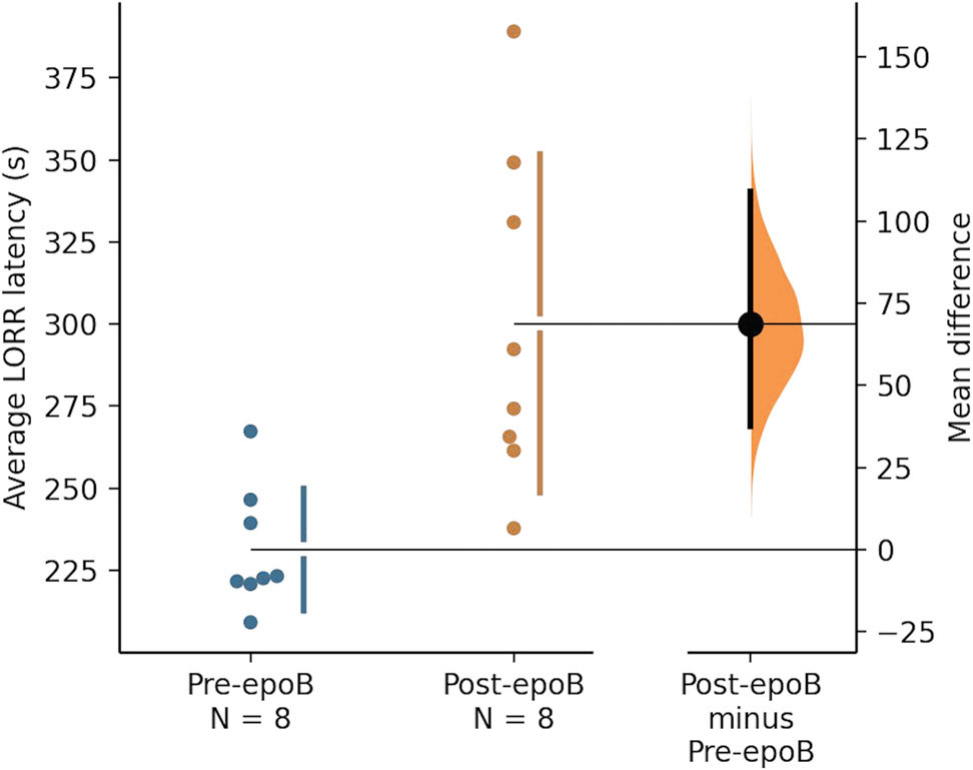
Average LORR latency increased after epoB injection. Average LORR latencies were calculated for each rat based on all anesthesia sessions prior to and after subcutaneous epoB injection (0.75 mg/kg). Injections during all other anesthesia sessions were vehicle only (50% saline/DMSO). The mean difference between pre-epoB and post-epoB LORR latencies is shown in the above Gardner–Altman estimation plot (Ho et al., 2019). Both groups are plotted on the left axes; the mean difference is plotted on a floating axis on the right as a bootstrap sampling distribution. The mean difference is depicted as a dot; the 95% confidence interval is indicated by the ends of the vertical error bar. The average LORR latency is 69 s higher in the post-epoB condition as compared with the pre-epoB average, and this difference is statistically significant (two-sided permutation *t* test; *N* = 8 rats; *p* = 0.0016^a^; Cohen's *d* = 1.9). The difference remained significant when we omitted eight outliers from the post-epoB dataset (*p* = 0.005^b^; *d* = 5.4). Superscripts on *p* values refer to rows of the statistical table, Table 2.

To statistically evaluate the overall effect of epoB on LORR latency across the group of eight rats, we compared LORR latencies measured before and after epoB injection. The average LORR latency increased by 69 s in the epoB condition, and this difference was significant (two-tailed permutation *t* test; *N* = 8 rats; *p* = 0.0016^a^; Fig. 2, Table 2). The magnitude of the difference corresponds to a standardized mean difference (Cohen's *d*) of 1.9, indicating a “large” normalized effect size. We identified eight outliers in post-epoB dataset, as values >75th percentile LORR latency plus 1.5*interquartile range, or <25th percentile value minus 1.5*interquartile range. Omitting outliers, the difference between the control and epoB conditions remained significant (*p* = 0.005^b^; *d* = 5.4).

**Table 2.** Statistical table showing confidence intervals on the mean difference between conditions for each comparison (*p* value) reported in the text

*P* value	Data structure	Test	95% Confidence interval
a	Empirical	Permutation *t* test	[37.4, 109] s
b	Empirical	Permutation *t* test	[12.8, 43.0] s
c	Normal	Unpaired 2-tail *t* test	[−10.5, 14.5] s/d
d	Normal	Unpaired 2-tail *t* test	[−28.7, 5.2] s/d
e	Normal	Unpaired 2-tail *t* test	[23.9, 113.6] s
f	Normal	Unpaired 2-tail *t* test	[72.1, 145.3] s
g	Normal	Paired 2-tail *t* test	[29.4, 50.5] s
h	Normal	Unpaired 2-tail *t* test	[23.3, 72.2] s

### EpoB-induced LORR latency increase is not accounted for by rats’ developing tolerance to isoflurane

**Figure 3.**
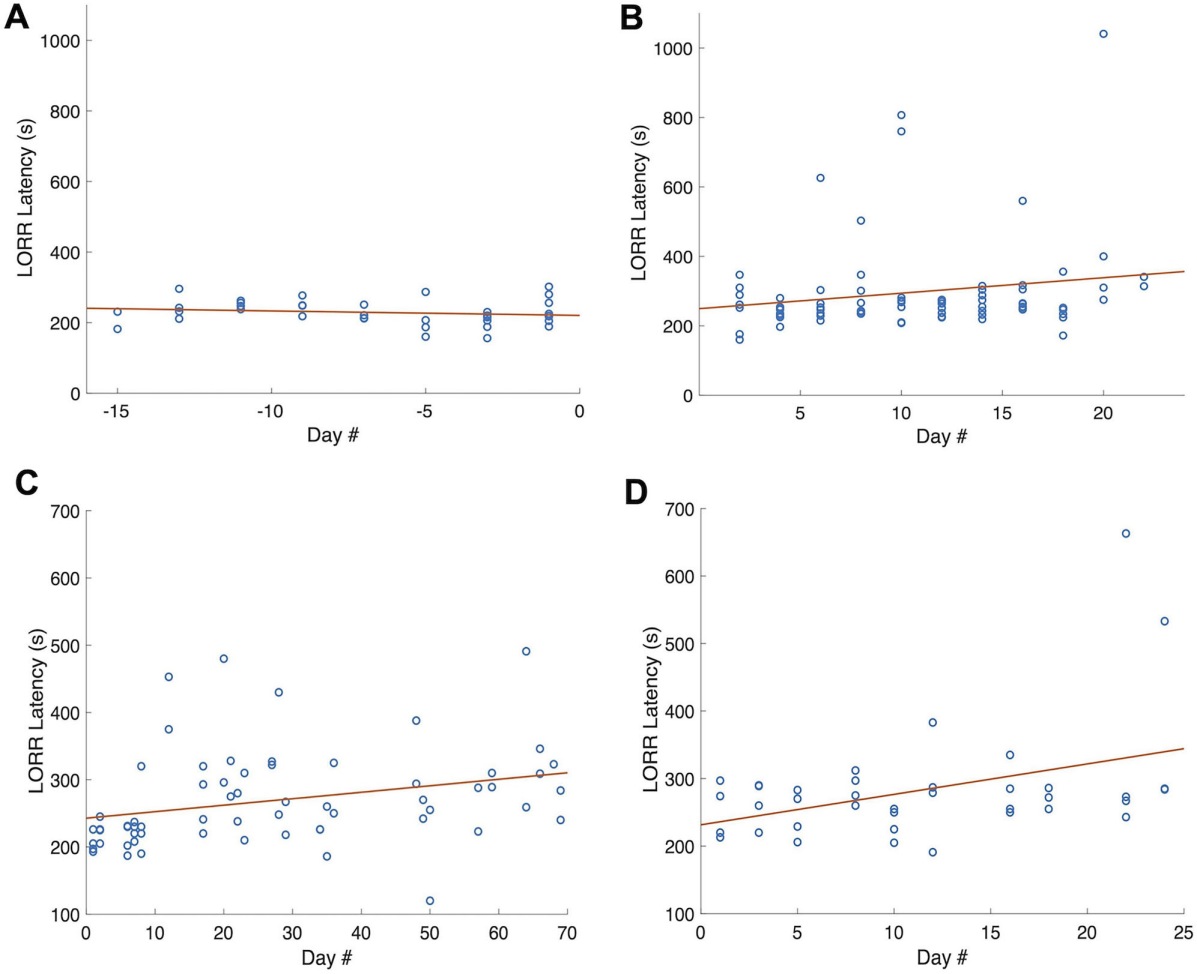
LORR latency time courses, for pre-epoB sessions (***A***), post-epoB sessions (***B***) for the main experimental group (*N* = 8 rats), and for another group of four “tolerance” rats not exposed to epoB (***C***, ***D***). Panel ***C*** shows the initial time course for the tolerance group, and panel ***D*** shows results from additional sessions conducted after a 41 d hiatus. Each open circle represents the LORR latency measured for one rat during one anesthesia session. Based on linear fits (solid lines), LORR latencies decreased on average by 1.3 s/d for the pre-epoB experiments (***A***) and increased by 4.5 s/d for the post-epoB sessions (***B***), 0.97 s/d for the tolerance group (***C***), and 4.5 s/d for the tolerance group after a 41 d pause in anesthesia sessions (***D***). The 95% confidence limits and average intervals between sessions for each of these series of experiments are shown in Table 3.

The increased LORR latency we measured in the epoB condition could be due to the effect of the epoB treatment. However, since the epoB sessions were all conducted after the control sessions, the increased LORR latencies we observed might also be accounted for by rats’ increasing resistance (tolerance) to isoflurane developed over repeated sessions (Chalon et al., 1983). To assess this possibility, we plotted LORR latency as a function of session in the pre-epoB period (Fig. 3*A*). A linear fit to these data resulted in a slope of −1.3 s/d, meaning an average LORR latency decrease of 1.3 s/d in the context of isoflurane anesthesia sessions taking place on average once every 3.1 d (Table 3). This decrease cannot account for the increase in average LORR latency shown in Figure 2.

**Table 3.** Linear estimates of tolerance development due to repeated anesthesia sessions, in the main experimental group before and after epoB injection, and the tolerance group before and after a 41 d pause in anesthesia sessions

Groups	Slope (s/d)	95% CI	*N*	*n*	Avg Int (*d*)	SD
Main pre-epoB	−1.3	(−3.43, 0.90)	8	36	3.1	1.8
Main post-epoB	4.5	(−0.20, 9.11)	8	76	2	0
Tolerance pre-pause	0.97	(0.29, 1.6)	4	63	4.6	3.9
Tolerance post-pause	4.5	(1.57, 7.45)	4	38	2.65	1.0

The slope column shows the slope of the linear fit to the LORR latency data in seconds per day. CI shows the 95% (3-sigma) confidence interval on each slope, *N* is the number of rats, and *n* is the number of sessions included in the estimate; Avg Int is the average interval between sessions in days, and SD is the standard deviation of the interval data.

However, this consideration is inconclusive because the interval between sessions in the post-epoB period was 2 d (SD, 0; Table 3), which is more frequent than in the pre-epoB sessions (Fig. 3*A*, Table 3). The tolerance effect might plausibly be larger in the condition with more frequent sessions. In order to construct a tolerance effect (slope) estimate when anesthesia sessions are repeated after an interval of 2 d, we identified pairs of sessions in the same rat separated by 2 d and calculated the LORR latency difference between each ordered pair. Averaging these latency differences (in seconds) across rats and dividing by the 2 d interval resulted in a 2 d interval slope estimate of 2.5 s/d [based on *n* = 20 session-pairs (i.e., intervals) in eight rats]. Although this slope estimate was not significantly different from zero slope (*t* test; *n* = 20 intervals; *p* = 0.7^c^; no significant tolerance effect), conservatively using this slope to estimate the tolerance contribution to the difference we observed between pre-epoB and post-epoB sessions results in a predicted tolerance-related increase in LORR latency of 2.5 s/d*16 d = 40 s. This is not enough to plausibly account for the 69 s average LORR latency increase we observed in the post-epoB period as compared with the pre-epoB period.

Moreover, we also evaluated potential tolerance effects in a separate group of four rats over a longer period of time, as shown in Figure 3*C*. In this case the 2-d-interval slope analysis resulted in a negative slope estimate of −11.5 s/d. This estimate also failed to be significantly different from zero but was a stronger trend [*p* = 0.1^d^; *n* = 36 session-pairs (i.e., intervals) in four rats] than the estimate from the pre-epoB period in the main experimental group. This supports that LORR latencies can actually drop across repeated sessions if the intersession interval is relatively short. Obviously, a drop in LORR latencies could not account for the increase we saw in the post-epoB condition. This result also supports that our estimate of 2.5 s/d of increased LORR latency across sessions separated by 2 d may be an upper bound on the true tolerance effect contribution to the change in LORR latency we saw after epoB injection.

Figure 3*C* also shows a linear fit to the LORR latency time course in the tolerance group, and Figure 3*D* shows a linear fit to LORR latency data taken from the same rats after a 41 d pause in anesthesia sessions. Before the pause, the linear fit resulted in an estimate of 0.97 s of increased LORR latency per day, in the context of anesthesia sessions taking place every 4.6 d on average. After the pause, the linear fit resulted in a slope of 4.5 s/d in the context of sessions taking place every 2.65 d on average (Table 3).

### Isoflurane tolerance effect persists for more than a month after halting anesthesia sessions

Figure 3*D* shows our LORR latency measurements taken from the tolerance group after a 41 d hiatus from anesthesia sessions. A comparison of the initial sessions with the final sessions in the pre-pause period (Fig. 3*C*) shows that LORR latencies were significantly higher toward the end of that pre-pause period (mean difference, 69 s; unpaired *t* test; *n* = 12 early vs *n* = 9 late sessions; *p* = 0.009^e^). This difference remained significant after omitting one outlier (mean difference, 109 s; *n* = 12 vs *n* = 8 sessions; *p* = 0.00019^f^).

The average LORR latency after the hiatus was significantly higher than the average LORR latency in the same animals’ first anesthesia sessions (mean difference, 40 s; paired *t* test; *n* = 12 earlier vs 12 later sessions; *p* = 0.0017^g^), supporting that the increased LORR latencies that developed over the initial series of anesthesia sessions persisted after more than a month without exposure to isoflurane. This difference remained significant after omitting one outlier session (mean difference, 48 s; *n* = 8 earlier vs 12 later sessions; *p* = 0.009^h^).

### Neither rat weight nor age predicts LORR latency

**Figure 4.**
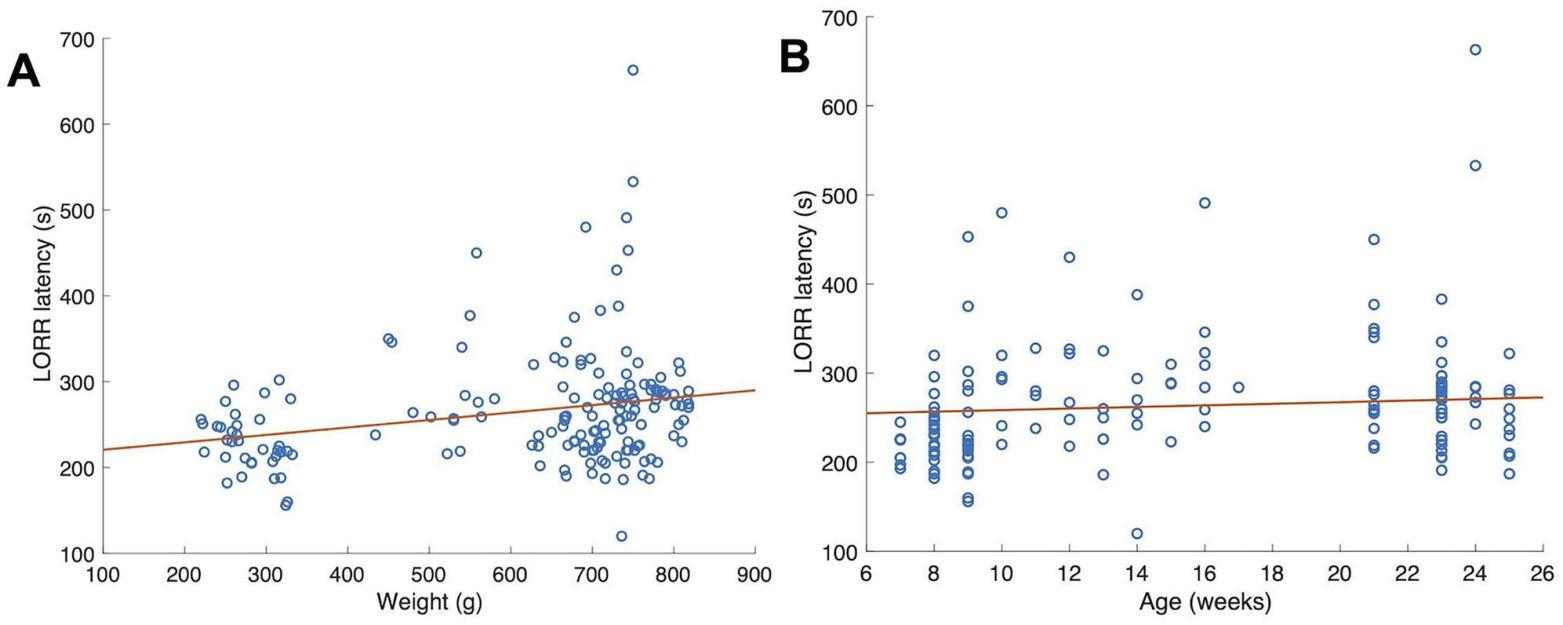
Relationship between LORR latency and rat weights (***A***) and age (***B***). Data collected from 59 anesthesia sessions in the no-epoB condition, from *N* = 12 rats. Linear fits to these data resulted in correlation coefficient squared (*R*^2^) of 0.058 for the weight fit and 0.029 for the age fit.

Our dataset also afforded us the opportunity to examine the potential relationship between LORR latencies and the weights and ages of our rats. Figure 4*A* shows LORR latencies of *N* = 12 rats in the no-epoB condition plotted against each rat's weight on the day of the LORR latency measurement. Figure 4*B* shows the same data plotted against each rat's age on the day of the LORR latency measurement. LORR latency was very weakly correlated with rat weight and age.

## Discussion

Our main finding was that rats under the influence of the brain-penetrant MT–stabilizing drug epoB took longer to become unconscious under isoflurane, as measured by LORR latency, compared with the control condition (Fig. 2). We used only male rats to avoid potentially confounding interactions with estrous phase in females. Note that we arranged for researchers taking the LORR latency measurements to be blind to the rats’ drug conditions even though the epoB condition came after the control condition for every rat (see Materials and Methods).

To determine whether the longer average LORR latency post-epoB might be accounted for in terms of our rats’ developing a tolerance to isoflurane over time rather than an effect of epoB, we estimated tolerance effects during the control period in our main experimental group of eight rats (Fig. 3). The tolerance effect we estimated in the control period of our main experiment was 1.3 s of decreased LORR latency per day, in the context of isoflurane sessions repeated on average once every 3.1 d. This effect obviously cannot account for an increase in LORR latency. However, we also considered the tolerance effect calculated from sessions separated by 2 d like the sessions in the post-epoB period of the main experimental group. This calculation resulted in a predicted tolerance-related increase in latency of ∼40 s in the post-epoB sessions. We emphasize that this is our most conservative estimate, given that our 2 d slope estimate in the separate tolerance group was negative like the overall slope of the pre-epoB data and that the 2 d slope we used to estimate a 40 s tolerance effect was not significantly different from zero (i.e., zero slope meaning no tolerance effect). In any case, 40 s is not enough to plausibly account for the 69 s average LORR latency increase we observed in the post-epoB as compared with the pre-epoB period.

Thus, since our main result cannot be accounted for by our rats’ developing tolerance to isoflurane, it strongly suggests that binding to MTs is one of the molecular mechanisms by which isoflurane causes unconsciousness in rats.

### Other work implicating MTs as a functional target of general anesthetics

Our findings are consistent with the report of partial anesthetic resistance in human surgical patients treated with taxane MT-stabilizing chemotherapy (Linganna et al., 2015) that was a primary motivation for our study in rats. Another study used MT-stabilizing agents epothilone D and discodermolide to directly implicate MTs as a functional target of general anesthetics in tadpoles (Emerson et al., 2013). They used anthracene and neurosteroid anesthetics rather than isoflurane, but they corroborate that perturbing MTs can contribute to unconsciousness.

In addition, detailed quantum chemical modeling of anesthetic and nonanesthetic molecules binding to the tubulin dimers that make up MTs found that anesthetic potency was predicted by the modeled disruption or failure to disrupt high-frequency vibrations inside MT protein subunits (Craddock et al., 2015, 2017). The latter study found a Meyer–Overton correlation for all eight anesthetics studied. This suggests possible unitary action of anesthetics in MTs.

### Other molecular mechanisms of isoflurane-induced unconsciousness

Isoflurane potentiates inhibitory GABA receptor activity in vitro (Hall et al., 1994; Garcia et al., 2010), and studies of mice with mutant GABA_A_ receptors concluded that GABA_A_ receptors contribute to LORR under isoflurane (the standard proxy for loss of consciousness). Isoflurane directly activates sleep-promoting neurons of the hypothalamic ventrolateral preoptic nucleus, and this contributes to causing unconsciousness (Moore et al., 2012; Kottler et al., 2013; Jiang-Xie et al., 2019). Further review of evidence bearing on these and other anesthetic targets may be found in Campagna et al. (2003); Hemmings et al. (2019); and Kelz and Mashour (2019).

### Longer-term and cumulative effects of exposure to volatile anesthetics

The average LORR latency was significantly higher in our tolerance group of rats after ∼70 d of anesthesia sessions repeated on average every 4.6 d, indicating that the rats did develop a tolerance to isoflurane in that context (Fig. 3*C*). Moreover, the average LORR latency remained significantly elevated after a 41 d pause in anesthesia sessions (Fig. 3*D*). This result shows that the tolerance developed over the course of 70 d of regular exposure to isoflurane did not revert to the shorter initial LORR latencies, after more than a month without isoflurane.

The tolerance effect we estimated in the main experimental group, from the control period of our epoB experiment, was negative, indicating a greater sensitivity after repeated sessions rather than a growing insensitivity (aka tolerance). This could occur, for example, if isoflurane remained in the animal's system long enough to contribute to hypnosis in subsequent anesthesia sessions after relatively short intervals without isoflurane exposure. These sessions were repeated with an average interval of 3.3 d between sessions. Although the average effect we measured was negative, the results were statistically consistent with no tolerance effect of isoflurane sessions repeated at that 3.3 d average interval.

Our estimate from a larger dataset collected over a longer period of time was that LORR latency increased on average 0.97 s/d in the context of 4.6 d on average between sessions. The fact that our slope estimate based only on pairs of sessions separated by 2 d was negative suggests that multiple competing processes of facilitation and depression of sensitivity to isoflurane with different characteristic time scales are active simultaneously, so that if intersession interval varies, complex nonlinear interactions may result.

In mice, continuous exposure to 0.15–0.3% isoflurane for 2–3 weeks was not found to produce a tolerance effect based on time to LORR (Smith et al., 1979). Another study found that exposure to 1% isoflurane given once a day for 10 consecutive days may produce a tolerance effect in mice as shown by longer LORR latencies (Chalon et al., 1983). On the other hand, mice repeatedly exposed to 4% isoflurane for 45 min at 3–4 d intervals for 3 weeks did not display significant differences in terms of nest building behavior, home cage activity, body weight, or corticosterone concentrations when compared with control mice (Hohlbaum et al., 2017). A more comprehensive understanding of the processes that determine sensitivity to repeated isoflurane exposure will require further study.

### Effects of weight or age on sensitivity to volatile anesthetics

#### Age

Our data did not reveal any significant relationship between latency to LORR and our rats’ ages. This contrasts with a study which found that older rats took significantly longer to emerge from isoflurane-induced unconsciousness than younger rats (Chemali et al., 2015). The younger rats in that study were 6–8 months old, and the older group was 24–26 months old. Our rats ranged in age from 7 to 25 weeks, i.e., ∼2–6 months old, which may not have been sufficient to show effects of advanced age. Moreover, effects of age in our experiments were confounded with potential effects of repeated isoflurane exposure and weight. In humans, anesthetic sensitivity generally increases with age, as indicated, for example, by the decrease in the minimum alveolar concentration of volatile anesthetics needed to abolish movement in response to a noxious stimulus, by ∼6% per decade (Kanonidou and Karystianou, 2007).

#### Weight

We found no significant relationship between LORR latencies and our rats’ varying weights. In humans, the surgically required concentration of inhalable anesthetics increases with body mass index, but the increase is small (Lemmens et al., 2008).

### How might anesthetic binding to MTs cause or contribute to unconsciousness? Competing theories of consciousness

It is conceivable that binding to MTs by volatile anesthetics could impair intracellular transport, which might disrupt synaptic transmission, which might reduce neural activity generally. In classical neuroscientific models of consciousness, reduced firing results in unconsciousness when it results in functional disconnection among cortical areas (He, 2024; Mashour, 2024), because consciousness in these models is understood in terms of functional connectivity (Dehaene et al., 1998; Sergent and Dehaene, 2004; Tsuchiya et al., 2015; Mashour et al., 2020) or classical integrated information (Tononi, 2008; Tononi et al., 2016; Albantakis et al., 2023) among neurons, cortical areas, or parts of the system. These theories do not at present predict specific molecular targets of anesthetics.

In contrast, another class of quantum model understands consciousness in terms of physical vibrational states of MTs in neurons (S. Hameroff and Penrose, 1996, 2014; Craddock et al., 2017; S. Hameroff, 2022). In this picture, quantum dipole vibrations in MTs inside neurons mediate consciousness, modulate functional connectivity, and regulate neuronal firing. Anesthetics dampen the quantum dipole oscillations and cause unconsciousness. That is, these models predict that volatile anesthetics interfere directly with the vibrational state of MTs which are understood as the immediate substrate of consciousness. As we noted above, detailed quantum chemical modeling studies support that anesthetic potency is predicted by the modeled disruption or failure to disrupt high-frequency vibrational states of MT proteins (Craddock et al., 2015, 2017).

Our results are potentially consistent with classical models of consciousness, but they represent a more stringent test of these MT-based models, because while the classical models are agnostic about MTs as a functional anesthetic target, the MT-based models specifically predict that directly disrupting the physical state of MTs should contribute to loss of consciousness.

Moreover, while the classical membrane-centered viewpoint appears to predict that perturbing MTs would primarily contribute to unconsciousness by interfering with synaptic transmission, so far no evidence points to MTs as the cause of isoflurane-induced disruption of synaptic transmission.

In different studies, isoflurane's effect reducing synaptic transmission has been shown to proceed primarily via its effect on syntaxin 1A (Herring et al., 2009) or via its effect inhibiting mitochondrial function (Zimin et al., 2018). This is not necessarily inconsistent with the classical model, since a change in the functional state of MTs could conceivably have an effect on synaptic proteins such as syntaxin 1A, for example. However, it does show that isoflurane's effect on synaptic transmission might be fully accounted for in terms of its interaction with other proteins besides MTs. That would represent additional support for the alternative hypothesis about how binding to MTs contributes to isoflurane-induced unconsciousness—that is, the hypothesis that binding to MTs interferes with the physical substrate of consciousness directly rather than by (or in addition to) indirect effects on neural membrane voltage and spiking activity.

In order for a quantum MT-based model of consciousness to be viable, (1) the putative conscious MT physical state must be able to interact with the membrane potential of neurons, where we know perceptual information is encoded and through which our actions (i.e., muscle activations) must be implemented, and (2) the quantum interaction must be able to span multiple neurons if it is to solve the perceptual binding problem. Actually both (1) and (2) have been experimentally verified by Anirban Bandyopadhyay's group (Singh et al., 2021b). In cultured neuronal networks, they found megahertz and gigahertz oscillations from dendritic–somatic MTs correlated with axonal firings in the same and other neurons better than did membrane potentials.

Criticizing the Penrose–Hameroff Orch OR theory, Tegmark (Tegmark, 2000) calculated MT decoherence would occur at 10^−13^ s, too fast for functional effects. However, Tegmark erred, using a key term for superposition separation distance of tens of nanometers (10^−8^ m) rather than femtometers (10^−15^ m) as stipulated in Orch OR. Hagan et al. (2002) corrected Tegmark and calculated 10^−5^–10^−4 ^s MT coherence time, potentially sufficient for Orch OR to influence neural processing. That paper also noted other inappropriate assumptions in Tegmark's analysis. These corrections were published in the same journal shortly after (Tegmark, 2000). Nevertheless, Tegmark's paper supported widespread skepticism that macroscopic or mesoscopic quantum effects could persist long enough at biological temperatures to be functionally relevant. The prediction of quantum coherence in MTs at high temperatures (i.e., room temperature) has now been experimentally verified (Saxena et al., 2020; Babcock et al., 2024).

A recent report describes theoretical predictions and experimental confirmation of superradiance—a fundamentally quantum effect (Dicke, 1954)—from MTs at room temperature that was enhanced as they were joined into larger structures (Babcock et al., 2024). Those authors cited theoretical work (Solano et al., 2017) in support of the “tantalizing possibility” that the physiological arrangement of MTs in axons could magnify the collective interaction “to an extremely long range.” Anirban Bandyopadhyay and colleagues explored resonant responses of MTs at frequencies varying over many orders of magnitude in “bare” MTs and in cultured neurons (Saxena et al., 2020; Singh et al., 2021a,b). They reported that electromagnetic waves transmitted to the MTs (acting like antennas) at MT resonant frequencies resulted in systematic modulation of the electric potential difference between two neighboring neurons. Their results support the conjecture of Babcock et al. that MTs can couple across neurons. Additionally, these results support that the MT vibrational state and resultant electromagnetic field can interact systematically with the membrane voltage that we know is closely related to mental function and behavior and again that this coupling can traverse the physical gap between neurons. In this context, it is also interesting to note a result demonstrating that isoflurane and sevoflurane were capable of interactions with quantum entangled photons, whereas nonhalogenated diethyl ether and all other sampled molecules were not, at the particular wavelength studied (Burdick et al., 2019). These experimental results corroborate the plausibility of the MT-based quantum physical model of consciousness and the hypothesis that isoflurane could contribute to abolishing consciousness by interacting with an entangled state of MTs.

The Orch OR MT-based consciousness theory was part of the Templeton World Charity Foundation program in Accelerating Research in Consciousness and produced the only confirmatory evidence among theories tested. The predictions were that (1) MT quantum effects would be shown at ambient temperature and (2) they would be inhibited by anesthesia (S. Hameroff, 2021). Kalra et al. (2023) showed that tryptophan fluorescence lifetimes propagated and persisted due to quantum exciton mechanisms, and these were dampened by two types of anesthetic, isoflurane and etomidate. Overall the Orch OR theory, in which MTs mediate anesthetic action, has more explanatory power, biological connection, and experimental validation than the classical theories.

### Future directions for experimentally testing the quantum MT-based consciousness model

Recent MRI (i.e., *nuclear* magnetic resonance imaging) studies appear to offer support for the general hypothesis that the physical substrate of consciousness in the brain must be a nontrivial macroscopic quantum state (C. M. Kerskens and Pérez, 2022; Pérez et al., 2023). The authors claim to demonstrate (1) the existence of a macroscopic quantum entangled state in the brain, (2) that this quantum brain state is likely related to consciousness and cognitive functioning, and (3) that this consciousness-related quantum state in the brain is capable of coupling to spin degrees of freedom in the nuclei of water molecules in the brain. They used an unconventional MRI protocol designed to isolate signals from entangled nuclear spins and observed MRI signals that closely resembled heartbeat-evoked potentials recorded with EEG. According to the authors, such signals should not be observable by MRI unless brain processes mediate entanglement among the nuclear spins in brain water. This implies the unknown brain process that mediates the entanglement is itself an entangled quantum state.

Because the fidelity of this putative spin-entanglement signal correlated with short-term memory performance (Pérez et al., 2023) and the presence or absence of the conscious state itself in sleep versus waking (C. M. Kerskens and Pérez, 2022), the authors concluded that the quantum brain processes are likely an important part of our cognitive and conscious brain functions. The interpretation in terms of entanglement has been challenged (Warren, 2023); however, that author did not propose an alternative classical account of the signal reported by Kerskens and Perez. Their response reaffirms their entanglement claim and provides further clarification of their argument (C. Kerskens and Pérez, 2023).

It remains to be seen whether a viable classical account of the signal observed by Kerskens and Perez will be proposed. However, their work is not the first experimental indication of functionally relevant coupling between nuclear spins and consciousness-related brain processes. Anesthesia experiments in mice demonstrated that isotopes of xenon with nuclear spin were less effective than isotopes without nuclear spin and that this difference could not be accounted for by other physical properties of the isotopes such as polarizability (Li et al., 2018). Nuclear spin is a physical property that in normal circumstances would not directly influence the chemical properties of the molecule or atom. Similarly, anesthetic experiments in fruit flies show that anesthetics perturb *electronic* spin states and that these spin responses are altered in anesthetic-resistant mutants (Turin et al., 2014), again supporting that a consciousness-related brain process can couple to spin degrees of freedom. Under the quantum MT picture, nuclear spin can affect anesthetic potency because anesthetics couple to electron clouds by quantum dipoles. Nuclear spin could actually promote electron entanglement by bosonic pair interactions (S. R. Hameroff, 2018). These experimental results may corroborate the MRI study above claiming the conscious brain process can interact with entangled nuclear spins.

Specific support for MTs as the actual physical substrate of consciousness in the brain could come from spectroscopic measurements of the brain tissue in living animals in anesthetized and awake conditions (Movasaghi et al., 2007), as suggested in Craddock et al. (2017). One or more spectroscopic peaks corresponding to the relevant resonance mode of the MTs in the conscious state would be expected to be shifted or reduced in amplitude during unconscious states produced by anesthesia or other means.

Along these lines, the Bandyopadhyay group has adapted methods from solid-state physics and quantum optics to noninvasively target known resonances of the MTs in living networks of neurons (Singh et al., 2021a,b; Ghosh et al., 2022). They have also developed a noninvasive extension of EEG, called DDG (“dodecanogram”), for measuring very high-frequency electromagnetic signals—some putatively originating from MT resonances internal to neurons—emitted from the scalps of awake humans (Singh et al., 2023, 2024). These recent technical developments support the hope that “some who are standing here will not taste death before they see” conclusive experimental tests of the quantum consciousness hypothesis.

## Synthesis

Reviewing Editor: Christine Portfors, Washington State University Decisions are customarily a result of the Reviewing Editor and the peer reviewers coming together and discussing their recommendations until a consensus is reached. When revisions are invited, a fact-based synthesis statement explaining their decision and outlining what is needed to prepare a revision will be listed below. The following reviewer(s) agreed to reveal their identity: Stuart Hameroff.

The reviewers agree that this is an important study. It is a straightforward study that adds important evidence for anesthetic action on microtubules, and can perhaps help resuscitate anesthesia mechanism research. The reviewers agree that there are issues that if addressed, could make the manuscript much stronger.

Specific comments:

Reviewer 1

1) There was insufficient blinding (videorecording with blinded observers would have been best, since the control and test compounds were always in the same order).

2) There were no data reported on emergence from the anesthetic, which would also provide insight in the effects of EpoB on anesthetic sensitivity. It is unclear why this is the case.

3) There are no data/discussion regarding off-target arousal effects of EpoB.

4) Discussion of the literature is biased. For example, the authors emphasize (in Abstract) with italics that quantum theory of consciousness/anesthesia predicted the role of microtubules but fail to note that part of the motivation for the role of microtubules in the quantum theory to begin with was their possible involvement in anesthetic mechanisms, which dates back to at least the 1960s (in other words, it was postdicted). As another example, the authors note (in Introduction) the change in blood pressure and opiate delivery with taxanes in the 2015 clinical study, but fail to mention that there was no change in heart rate or anesthetic delivery.

Reviewer 2:

This interesting paper can contribute toward understanding anesthetic action and consciousness. But several important scientific and historical concepts would be helpful for context. These are:

1) The Meyer-Overton correlation (and additivity of MAC/potency) suggests a unitary action of all anesthetics on one target protein.

2) Particular membrane receptor and ion channel proteins were ruled out as possible unitary targets by exhaustive studies culminating in Eger et al (2008). However tubulin/microtubules were not ruled out and remain a candidate for a unitary site of anesthetic action (which the current work supports),

3) The Orch OR theory proposes anesthesia directly blocks quantum effects in microtubules necessary for consciousness

4) Anesthetics act by weak, non-specific quantum interactions between induced dipoles.

So some suggestions:

Title

Microtubule-stabilizer epothilone B delays unconsciousness in under isoflurane in rats

Reviewer

Some will argue whether rats are conscious (I believe they are) and the words used in anesthesia literature to describe what 'goes away' are 'mobility' or 'purposeful behavior'. I think 'unconsciousness' is OK if linked to anesthesia.

Also I'm not sure how many readers will recognize that isoflurane is an anesthetic gas.

Maybe something like?

Microtubule-stabilizer epothilone B delays anesthesia-induced unconsciousness in rats

Abstract

Authors

Line 2

'Volatile anesthetics are currently believed to cause unconsciousness by acting on a combination of different molecular targets including neural ion channels and receptors, mitochondria, synaptic proteins, and cytoskeletal proteins; but the precise combination remains unclear.'

Reviewer

Tubulin/microtubules remain a potential site for unitary action. Maybe say:

:

'Volatile anesthetics are currently believed to cause

unconsciousness by acting on one or more molecular

targets, including neural ion channels and receptors,

mitochondria, synaptic proteins, and cytoskeletal proteins.

Next in abstract:

Line 4

Volatile anesthetics bind to cytoskeletal microtubules (MTs) and disrupt

their stability which could contribute to causing unconsciousness, for example by interfering with synaptic vesicle transport within neurons.

This possibility...

Reviewer

We don't know for sure the isoflurane caused destabilization, nor that destabilization causes unconsciousness. Vesicle transport probably too slow for consciousness.

And evidence suggests anesthesia dampens quantum optical effects in MTs which are implicated in consciousness. So also possible slight destabilization dampens quantum effects by disturbing crystalline alignment. See further below.

S better to say:

Line 4

Volatile anesthetics bind to cytoskeletal microtubules (MTs), disrupt

their stability and dampen their quantum optical effects,

both mechanisms potentially contributing to causing unconsciousness.

This possibility is supported by the finding that taxane....

Lines 15, 16

Authors

Our results suggest that destabilization of microtubules is one of the

mechanisms by which isoflurane causes unconsciousness in rats.

Reviewer

You haven't shown destabilization which is not necessarily the mechanism. The simplist explanation for your results is that the epothilone B blocked access to, or partially occupies the isoflurane binding pocket (as taxane does). Would suggest:

Our results suggest that binding of the anesthetic gas isoflurane to microtubules causes

unconsciousness and loss of purposeful behavior in rats (and presumably humans and other animals).

Authors

Line 17

This finding is consistent with classical models of consciousness as a functional state of electrical signaling among networks of neurons.

Reviewer

Not really. I don't think this statement is true. Classical models say microtubules have no direct effect on neuronal membrane and synaptic signaling.

However Singh et al (2021) show

microtubule activities do regulate axonal firing, for example, overriding membrane potentials. https://journals.physiology.org/doi/full/10.1152/jn.00478.2020

'Classical models' of consciousness ignore neuronal interiors and microtubule processing.

I'd suggest just say

Line 18

This finding is consistent with and predicted by models that posit consciousness as a property of quantum physical states of neural microtubules.

Line 20

Significance Statement

Authors

Our study establishes that destabilization of intracellular microtubules is one of the molecular mechanisms by which the inhalable gas isoflurane induces unconsciousness in rats....

Reviewer

Your study didn't demonstrate destabilization of microtubules, which isn't necessarily the mechanism anyway.

Better would be:

Our study establishes that anesthetics act on intracellular microtubules as the mechanism, or one of the mechanisms, by which the inhalational anesthetic gas isoflurane induces unconsciousness in rats.

Good from there..

Page 2

Line 28

Authors

Introduction

The functionally relevant molecular targets for causing unconsciousness are

believed to be a combination of neural ion channels and receptors, mitochondria, synaptic proteins, and cytoskeletal proteins. However the precise combination remains unknown.

Reviewer

As above, Better to say

Introduction

The functionally relevant molecular targets for causing unconsciousness are

believed to be one, or a combination of neural ion channels and receptors, mitochondria, synaptic proteins, and cytoskeletal proteins.

I suggest

Line 39

....Hoogenraad, 2015).

Add something like:

Microtubules have also been proposed to process information, encode memory and mediate consciousness (Hameroff and Watt, 1982; Hameroff &Penrose,1996; 2014; Hameroff, 2022)

equency vibrations in terahertz 1012 Hz, infra-red and visible photons) inside MT protein subunits (Craddock et al, 2015; 2017).

I'd suggest also something like:

Although molecular modeling rather than biological experiments, Craddock et al (2017) suggests a Meyer-Overton correlation for all 8 anesthetics studied. This suggests possible unitary action of anesthetics in microtubules.

Those authors also noted....

line 281

How might disruption of MT stability contribute to unconsciousness? Competing theories of consciousness

As per previous, for the heading I'd suggest something like

How might anesthetic binding to microtubules cause or contribute to unconsciousness? Competing theories of consciousness

The first sentence 'doubles down' on disruption of MT stability as cause of unconsciousness. I don't see it in Pan et al, 2007, or anywhere else. Should balanced with other possibiities.

Line 295

The references in parentheses should include

Hameroff and Penrose, 1996; 2014; Hameroff, 2022)

Line 296 I'd suggest something like

In this picture, quantum dipole vibrations in microtubules inside neurons mediate consciousness, functional connectivity and regulate neuronal firing. Anesthetics dampen the quantum dipole oscillations and cause unconsciousness.

Line 297 These models allow for the possibility.....

Line 325

perceptual binding problem. Actually both (a) and (b) have been experimentally verified by Anirban Bandyopadhyay's group (Singh et al 2021)

https://pubmed.ncbi.nlm.nih.gov/33881910/

In cultured neuronal networks they found megahertz and gigahertz oscillations from dendritic-somatic microtubules correlated with axonal firings in the same, and other neurons better than did membrane potentials.

Moreover...

Line 326

...to be functionally relevant. Criticizing the Penrose-Hameroff Orch OR theory, Tegmark (2000) calculated MT decoherence would occur at 10^-13 secs, too fast for functional effects. However Tegmark erred, using a key term for superposition separation distance tens of nanometers (10-^8 m) rather than femtometers (10^-15 m) stipulated in Orch OR. Hagan et al (2002) corrected Tegmark and calculated 10-6 secs MT coherence time, sufficient for Orch OR and experimentally verified in MTs (Saxena et al, 2020), and published in the same journal a year later than Tegmark's incorrect attack.

Hagan et al

https://journals.aps.org/pre/abstract/10.1103/PhysRevE.65.061901

Line 345

Singh et al (2024) showed entanglement in angular momentum between two separated MTs at ambient temperature.

Line 374

...nuclear spin affects anesthetic potency because anesthetics couple to electron clouds by quantum dipoles.

But nuclear spin can promote electron entanglement by

'radical pair'

Interactions (Hameroff, 2018)

https://pubmed.ncbi.nlm.nih.gov/29794803/

Line 384, before line 385

The Orch OR theory was part of the Templeton World Charity Foundation (TWCF) program in Accelerating Research in Consciousness, and produced the only confirmatory evidence among theories tested. The predictions were that 1) MT quantum effects would be shown at ambient temperature, and 2) they would be inhibited by anesthesia (Hameroff, 2021). Kalra et al (2024) showed that tryptophan fluorescence lifetimes propagated and persisted due to quantum exciton mechanisms. And they were dampened by two types of anesthetic, isoflurane and etomidate (Kalra et al, 2024)

https://pubs.acs.org/doi/10.1021/acscentsci.2c01114

Overall the Orch OR theory, in which MTs mediate anesthetic action, has more explanatory power, biological connection, and experimental validation than classical theories combined.

## Author Response

We sincerely thank both reviewers for their substantial suggestions that will certainly strengthen our paper. We also appreciate the comment that our study is "important" and may even "help resuscitate anesthesia mechanism research." Below we respond to each specific reviewer comment.

Reviewer 1:

1. Insufficient blinding. Since the "control and test compounds were always in the same order," we made somewhat elaborate arrangements to ensure that researchers taking the LORR latency data were blind to the drug condition and even to what stage of the study we were at. All researchers were trained to use the identical procedure for determining LORR latency, and at least two researchers (both blind) were present for every experiment. This is noted in our revised Methods section. We did not videotape experimental sessions though.

2. Missing emergence data. Our experimental design involved injections during every experimental session. To avoid stressing the rats before taking LORR measurements, which would likely introduce large uncontrolled variability into the data, we gave injections after isoflurane induction. This preserved integrity of the LORR data but unfortunately did introduce large variability into the emergence (RORR) data, which prevented our drawing any firm conclusions from those data. This is noted in our revised Methods section.

3. Potential off-target arousal effects of EpoB. We thank Reviewer 1 for catching this oversight. Our revised manuscript addresses this issue in the Methods section.

4. Biased literature discussion. Given that Reviewer 1 and Reviewer 2 reached consensus, we understand this comment to mean that our discussion was biased in favor of the classical membrane-voltage-based picture as opposed to the quantum microtubule hypothesis. Taking Reviewer 2's comments into consideration as well, we have revised the Abstract, Introduction, and Discussion sections to correct the bias. In particular we have revised the Introduction and Discussion to clarify the long history of microtubules as a candidate target of anesthetics.

As another example, the authors note (in Introduction) the change in blood pressure and opiate delivery with taxanes in the 2015 clinical study, but fail to mention that there was no change in heart rate or anesthetic delivery. We have revised the Introduction to correct this omission.

Reviewer 2:

1. Missing scientific context. The reviewer brought up 4 specific points that help to motivate our study. In retrospect, our submitted introduction was a misguided attempt to be conservative. We have revised our Introduction to include all four of these suggested points (Meyer-Overton; Eger 2008 left microtubules viable, Orch OR, and non-specific anesthetic quantum interactions).

2. Title. We appreciate the suggestion to make our title more accessible to a wider readership, so we have adopted Reviewer 2's suggested title in the revised manuscript: "Microtubule-stabilizer epothilone B delays anesthetic-induced unconsciousness in rats" 3. Abstract. One or more. We are happy to take the suggested sentence: "Volatile anesthetics are currently believed to cause unconsciousness by acting on one or more molecular targets, including neural ion channels and receptors, mitochondria, synaptic proteins, and cytoskeletal proteins." We have also incorporated the point that "Tubulin/microtubules remain a potential site for unitary action" into the revised Introduction.

4. Abstract. Line 4 Better to say: "Volatile anesthetics bind to cytoskeletal microtubules (MTs), disrupt their stability and dampen their quantum optical effects, both mechanisms potentially contributing to causing unconsciousness. ...This possibility is supported by the finding that taxane...." Done, and we omitted the dubious reference to stability disruption.

5. Abstract. Lines 15, 16. Destabilization vs blocking binding. We agree with the Reviewer's point: You haven't shown destabilization which is not necessarily the mechanism. The simplest explanation for your results is that the epothilone B blocked access to, or partially occupies the isoflurane binding pocket (as taxane does). Would suggest: "Our results suggest that binding of the anesthetic gas isoflurane to microtubules causes unconsciousness and loss of purposeful behavior in rats (and presumably humans and other animals)." We agree. This suggestion is adopted in our revised manuscript, and we have revised all references to stability disruption in the paper.

6. Abstract. Line 18 just say "This finding is consistent with and predicted by models that posit consciousness as a property of quantum physical states of neural microtubules." Done (we omitted "is consistent with and" because it is redundant). We have also incorporated the salient point from Singh et al (2021) into our revised Introduction.

7. Significance Statement. Line 20. Better would be: "Our study establishes that anesthetics act on intracellular microtubules as the mechanism, or one of the mechanisms, by which the inhalational anesthetic gas isoflurane induces unconsciousness in rats." Done.

8. Introduction. Line 28. As above, better to say: "The functionally relevant molecular targets for causing unconsciousness are believed to be one, or a combination of neural ion channels and receptors, mitochondria, synaptic proteins, and cytoskeletal proteins." Done.

9. Introduction. Line 39. After ...Hoogenraad, 2015). I suggest add something like: "Microtubules have also been proposed to process information, encode memory and mediate consciousness (Hameroff and Watt, 1982; Hameroff &Penrose,1996; 2014; Hameroff, 2022)." We have adopted this sentence in the revised Introduction.

Discussion: I'd suggest also something like: "...vibrations in terahertz 1012 Hz, infra-red and visible photons) inside MT protein subunits (Craddock et al, 2015; 2017). Although molecular modeling rather than biological experiments, Craddock et al (2017) suggests a Meyer-Overton correlation for all 8 anesthetics studied. This suggests possible unitary action of anesthetics in microtubules." We have adopted this suggestion in the revised Discussion.

10. Discussion. Line 281 for the heading I'd suggest something like "How might anesthetic binding to microtubules cause or contribute to unconsciousness? Competing theories of consciousness." Done. The first sentence 'doubles down' on disruption of MT stability as cause of unconsciousness. I don't see it in Pan et al, 2007, or anywhere else. Should be balanced with other possibilities. We agree and have revised this section to correct our mistake. We now describe disruption of MT stability as "conceivable" rather than "plausible." 11. Discussion. Line 295 The references in parentheses should include (Hameroff and Penrose, 1996; 2014; Hameroff, 2022). Now they do.

12. Discussion. Line 296 I'd suggest something like "In this picture, quantum dipole vibrations in microtubules inside neurons mediate consciousness, functional connectivity and regulate neuronal firing. Anesthetics dampen the quantum dipole oscillations and cause unconsciousness. ...These models allow for the possibility..." Very good; and done.

13. Discussion. Line 325 after "perceptual binding problem." "Actually both (a) and (b) have been experimentally verified by Anirban Bandyopadhyay's group (Singh et al 2021) "In cultured neuronal networks they found megahertz and gigahertz oscillations from dendritic-somatic microtubules correlated with axonal firings in the same, and other neurons better than did membrane potentials. Moreover..." And Line 326 after "to be functionally relevant." Criticizing the Penrose-Hameroff Orch OR theory, Tegmark (2000) calculated MT decoherence would occur at 10^-13 secs, too fast for functional effects. However Tegmark erred, using a key term for superposition separation distance tens of nanometers (10-^8 m) rather than femtometers (10^-15 m) stipulated in Orch OR. Hagan et al (2002) corrected Tegmark and calculated 10-6 secs MT coherence time, sufficient for Orch OR and experimentally verified in MTs (Saxena et al, 2020), and published in the same journal a year later than Tegmark's incorrect attack." We are pleased to include both of these excellent points in our revised Discussion.

14. Discussion. Line 345. Singh et al (2024) showed entanglement in angular momentum between two separated MTs at ambient temperature. We are unable to find this article. The closest we found was Sahoo, Singh et al 2023 "A third angular momentum of photons." We searched for articles that cite this one but did not find a microtubule entanglement paper. This paper is amazing but since it is about artificial nanowires rather than microtubules we don't think it is the study you are referring to. We contacted Anirban Bandyopadhyay and he said that the paper about two entangled microtubules is under review. We hope we will have the opportunity to add a sentence to cite this paper if it is in press by the time we are reviewing proofs for our study.

15. Discussion. Line 374 "...nuclear spin affects anesthetic potency because anesthetics couple to electron clouds by quantum dipoles. But nuclear spin can promote electron entanglement by 'radical pair' Interactions (Hameroff, 2018)." Thank you, we have incorporated this into our revised Discussion. On reviewing this paper we don't find reference to "radical pair" interactions, so we think you are talking about "bosonic pair" interactions that were described in this paper.

16. Discussion. Line 384, before line 385. "The Orch OR theory was part of the Templeton World Charity Foundation (TWCF) program in Accelerating Research in Consciousness, and produced the only confirmatory evidence among theories tested. The predictions were that 1) MT quantum effects would be shown at ambient temperature, and 2) they would be inhibited by anesthesia (Hameroff, 2021). Kalra et al (2024) showed that tryptophan fluorescence lifetimes propagated and persisted due to quantum exciton mechanisms. And they were dampened by two types of anesthetic, isoflurane and etomidate (Kalra et al, 2024). Overall the Orch OR theory, in which MTs mediate anesthetic action, has more explanatory power, biological connection, and experimental validation than classical theories combined." We are happy to include this relevant context in our revised Discussion. However, since it pertains to history we decided to place it before the Future Directions section.

To accommodate these changes within the 3000-word limit for the Discussion, we cut some other text, as noted in the changes-tracked document.

We also revised our visual abstract to remove the misleading image of exploding microtubules.
